# Another Drill Stock

**Published:** 1851-01

**Authors:** 


*Another Drill Stock.*—In the last number of the Journal we gave an engraving of a drill-stock recently invented by Dr. W. W. H. Thackston, of Va., and the October No. of the New York Dental Recorder contains an illustrated description of another gotten up by Mr. Chevalier, dental instrument manufacturer, of New York. The end of the instrument, in which the drill works, as well as the shaft, resembles the drill stock invented by Dr. Maynard of Washington, some years ago, but this is worked by a crank at the side of the instrument, above the handle, where the shaft terminates, while Dr. M's is moved by a nob at the end of the shaft, below the handle. With regard to the comparative merits of the two instruments, we are unable to decide, not having had an opportunity of giving Mr. Chevalier's a trial. Judging from the engraving, it is a very beautiful and ingeniously contrived instrument.

